# Mesoporous Silica Particles Retain Their Structure and Function while Passing through the Gastrointestinal Tracts of Mice and Humans

**DOI:** 10.1021/acsami.2c16710

**Published:** 2023-02-02

**Authors:** Muhammad Naeem Iqbal, Ghislaine Robert-Nicoud, Marina Ciurans-Oset, Farid Akhtar, Niklas Hedin, Tore Bengtsson

**Affiliations:** † Department of Materials and Environmental Chemistry, Stockholm University, StockholmSE-106 91, Sweden; ‡ Sigrid Therapeutics AB, Stockholm, Stockholm113 29, Sweden; § Division of Materials Science, Department of Engineering Sciences and Mathematics, Luleå University of Technology, LuleåSE-971 87, Sweden; ∥ Department of Molecular Biosciences, The Wenner-Gren Institute, Stockholm University, StockholmSE-106 91, Sweden

**Keywords:** mesoporous silica particles, biostability, gastrointestinal tract, protein adsorption, porcine pancreatic α-amylase

## Abstract

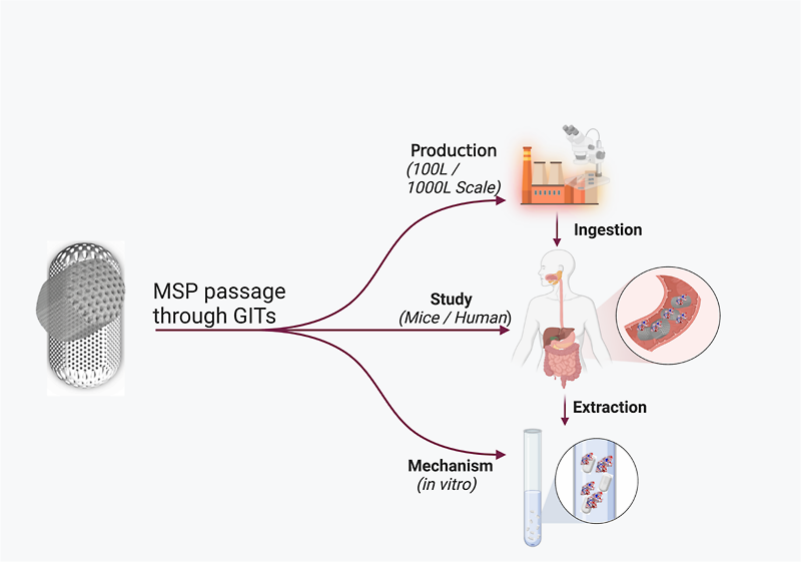

Mesoporous silica particles (MSPs) can be used as food additives, clinically for therapeutic applications, or as oral delivery vehicles. It has also been discussed to be used for a number of novel applications including treatment for diabetes and obesity. However, a major question for their possible usage has been if these particles persist structurally and retain their effect when passing through the gastrointestinal tract (GIT). A substantial breaking down of the particles could reduce function and be clinically problematic for safety issues. Hence, we investigated the biostability of MSPs of the SBA-15 kind prepared at large scales (100 and 1000 L). The MSPs were orally administered in a murine model and clinically in humans. A joint extraction and calcination method was developed to recover the MSPs from fecal mass, and the MSPs were characterized physically, structurally, morphologically, and functionally before and after GIT passage. Analyses with N_2_ adsorption, X-ray diffraction, electron microscopy, and as a proxy for general function, adsorption of the enzyme α-amylase, were conducted. The adsorption capacity of α-amylase on extracted MSPs was not reduced as compared to the pristine and control MSPs, and adsorption of up to 17% (w/w) was measured. It was demonstrated that the particles did not break down to any substantial degree and retained their function after passing through the GITs of the murine model and in humans. The fact the particles were not absorbed into the body was ascribed to that they were micron-sized and ingested as agglomerates and too big to pass the intestinal barrier. The results strongly suggest that orally ingested MSPs can be used for a number of clinical applications.

## Introduction

Mesoporous silica particles (MSPs) have pores of 2–50 nm and are researched for applications that include food science, therapeutics, and drug delivery.^1−3^ Ordered MSPs have tunable physical characteristics such as surface area, pore volume, pore size, morphology, crystallography, and particle size. The archetypical ones are those of the Santa Barbara Amorphous (SBA) and Mobil Composition of Matter (MCM) types.^4,5^ Their high internal surface areas and pore volumes also imply that they can circumvent the often-slow pharmacokinetics for oral formulations.^6−9^ These features give MSPs the ability to rapidly transport mass; for example, it is reported that the transport of ibuprofen through MSPs is very fast (with a mass transport coefficient of 1 × 10^–4^ s^–1^).^10^ The rapid mass transport of molecules and smaller macromolecules is appealing for related applications. The high enzyme adsorption capacity and their stability in MSPs are two of their appealing features that make them a suitable choice for biocatalytic support.^11^

Oral delivery is of importance for applications in foods and drugs as it is the most convenient and preferred route for administration.^6,12^ However, it is in general challenging to develop a safe and effective drug delivery system due to the harsh physiological environment of the gastrointestinal tract (GIT).^13^ In this context, we have shown that MSPs with mesopores above 7 nm are potential candidates for the treatment and prevention of metabolic syndrome and type-2 diabetes mellitus.^2,14−16^ It has been questioned if orally ingested micron-sized MSPs are suitable for therapeutic applications and if they can pass through the GIT without being systematically absorbed. At the end of the GIT motility, the particles would be secreted into the fecal mass. There is a lack of stability studies performed on orally ingested MSPs. Studies that have been performed ex situ using, for example, buffer incubation alone are likely not sufficient in assessing the usability of the MSPs as they do not fully mimic the GIT as it consists of a complex passageway involving numerous organs and environments. The transit time of the particles through the GIT is usually greater than a few hours but less than a week.^17^ It becomes critical to determine the biostability at the level of real exposure time of the mesoporous particles to the GIT for their long-term viability in therapeutic applications or as delivery vehicles for oral administration.

Previously, the degradation and biostability of particles of mesoporous silica have been studied primarily in in vitro systems and through intravenous injection in animal models. Some of these studies have found that particles in these systems degrade to some degree during the treatment.^18−21^ However, the used particles have not been sufficiently well characterized to allow for deriving correlations among structural details, function, and off-target effects. The importance of the internal specific surface area of MSPs is recognized, but the particle size and structural details of the surface region (skin effects) of the particles are also critical to the mass transport of molecules and macromolecules.^19,21−25^ Also, how to transfer an understanding of the degradation of particles from studies of injected delivery in different tissues to those of oral delivery is not obvious.

The biostability of particles of mesoporous silica depends on the particle size, surrounding media, and so forth.^26,27^ It appears that functionalized particles are more stable against biodegradation.^27^ To investigate if MSPs are stable during passage through the GITs, a set of experiments was performed, and the particles were studied before and after passing through the digestive tracts of mice and humans. The MSPs were synthesized at a controlled large scale and studied physically, structurally, morphologically, and functionally. The MSPs were tested for their ability to adsorb α-amylase—a carbohydrate digestive enzyme. We used this protein to test protein absorption as it is abundant in the GIT. It was demonstrated that the silica particles did not break down and retain their function after passaging through the GITs of mice and humans. The results are important because they indicate that MSPs can be used clinically in the rapidly expanding field of food additives, therapeutic applications, and oral delivery vehicles.^28,29^

## Experimental Section

### Material Synthesis

MSPs of the SBA-15 kind were synthesized according to previously reported methods.^14,16^ All the chemicals used for the synthesis of the MSPs were sourced from Sigma-Aldrich (MO, USA) and used without purification. The MSPs were synthesized at gram-scale and upscaled to the 100 and 1000 L scales. At the g-scale, 4 g of P123, a triblock copolymer (with average *M*_W_ = 5800 g/mol, EO_20_PO_70_EO_20_—Sigma-Aldrich product ID 435465), was dissolved in aqueous hydrochloric acid (37%—Sigma-Aldrich product ID 1003179200), with an acid concentration equivalent to 1.8 M, and a volume of 500 mL. After complete dissolution of the P123, 9 mL of a silica source, i.e., tetraethyl orthosilicate (TEOS—99%—Sigma-Aldrich product ID 333859), was added under vigorous stirring at 40 °C. The final molar ratio of the involved components was P123/TEOS/HCl/H_2_O: 0.0200:1.00:6.05:180.16. The water used was of a MilliQ grade. The reactive system was then kept static at this temperature for 24 h; after that, it was hydrothermally treated at a temperature of 100 °C for a time of 17–50 h. The mesostructured hybrid particles were then filtered, washed, and dried. Finally, these hybrid particles were subjected to calcination to remove the P123 in a muffle furnace at 550 °C (in the air) for 6 h dwelling using 10 h of ramping.^16^ Material was calcined in a batch process, pooled, and homogenized at the end.

Similar processes were used for the upscaling of the MSP synthesis. The material scaled up to the 100 L scale (MSP 1) was used in murine-based experimentation. MSP 2 scaled up to a 1000 L scale was used for the human study.^14^ When scaling up from 100 to 1000 L, minor process changes were made for bulk material handling and release of the MSPs for a human trial. For both batches, an average pore size of 10 ± 1 nm, minimum surface area of 600 m^2^/g, and pore volume of 0.8 cm^3^/g were part of the design and property criteria. The synthesis was performed in a glass-lined Hastelloy reactor of 1200 L capacity. All the production, release, packaging, and transferring of the material was performed under the GLP/GMP guidelines.

### Material Characterization

Physical characteristics of the MSPs were determined with analyzes of N_2_ sorption, low-angle X-ray diffraction (LAXRD), scanning electron microscopy (SEM), and transmission electron microscopy (TEM). Further details on the instrumentation and preparation procedures are presented in the Supporting Information. N_2_ sorption curves for MSP 1/2-Pristine are provided in Figures S-2 and S-3. SEM, TEM, and LAXRD analysis for MSP 1/2-Pristine are listed in Figures S-5–S-7, respectively.

#### Administration of MSPs in Mice and Related Work-Up of Feces

Briefly, the MSP 1 batch was weighed and dried at 120 °C. On the next day, the MSP 1 was weighed again to obtain precisely the MSP 1 weight. MSP 1 was mixed with high fat and high carb (HFHC) diet (bought commercially with diet ID-D12451, Research Diets Inc., USA) at 4% (weight/weight) and thoroughly homogenized in a food blender. Afterward, HFHC mixed with MSP 1 was repalletized by hand. A dosing of 4% was selected based on the reported no-observed-adverse-effect-level.^16^ A control group receiving the HFHC diet without any MSPs was part of the study. The animals were 9-week-old male mice (C57BI/6N, Scanbur, Sweden) and were single caged. Animals were kept at 12 h:12 h light/dark cycle at a thermos-neutral temperature of 30 °C and 5% humidity. Animals had free access to their diet and water during the whole experiment. Food intake was measured weekly. Fecal samples were collected during weeks 6 and 7.^15^ Samples were dried at room temperature before weighing and storing at −20 °C until the analysis and extraction procedure was performed. A pooled sample was prepared with an equal weight of feces from each mouse fed with MSP 1. This sample was further freeze-dried (FD) and homogenized before any analysis, calcination, and extraction procedures. The designed study was approved by the animal ethics committee of the North Stockholm Region (Dnr N240/14).

#### Oral Administration of MSPs in Humans and Related Work-Up of Feces

Briefly, the oral administration was conducted according to the good clinical practice (GCP), ISO14155, conformed to the Declaration of Helsinki, and was registered in the clinical trials registry (Clinical Trial Registration: NCT03823027). The participants were instructed to take 3 g of MSPs, mixed in a glass of water, three times per day before meal. The participants were further advised to have a normal dietary intake. According to the study design, the participants collected the feces samples at the week 6-time point after continual consumption. The feces samples were kept at a temperature of −80 °C before any treatment and analysis. Feces from all the participants who ingested batch “MSP 2” were pooled and FD.

**Scheme 1:**
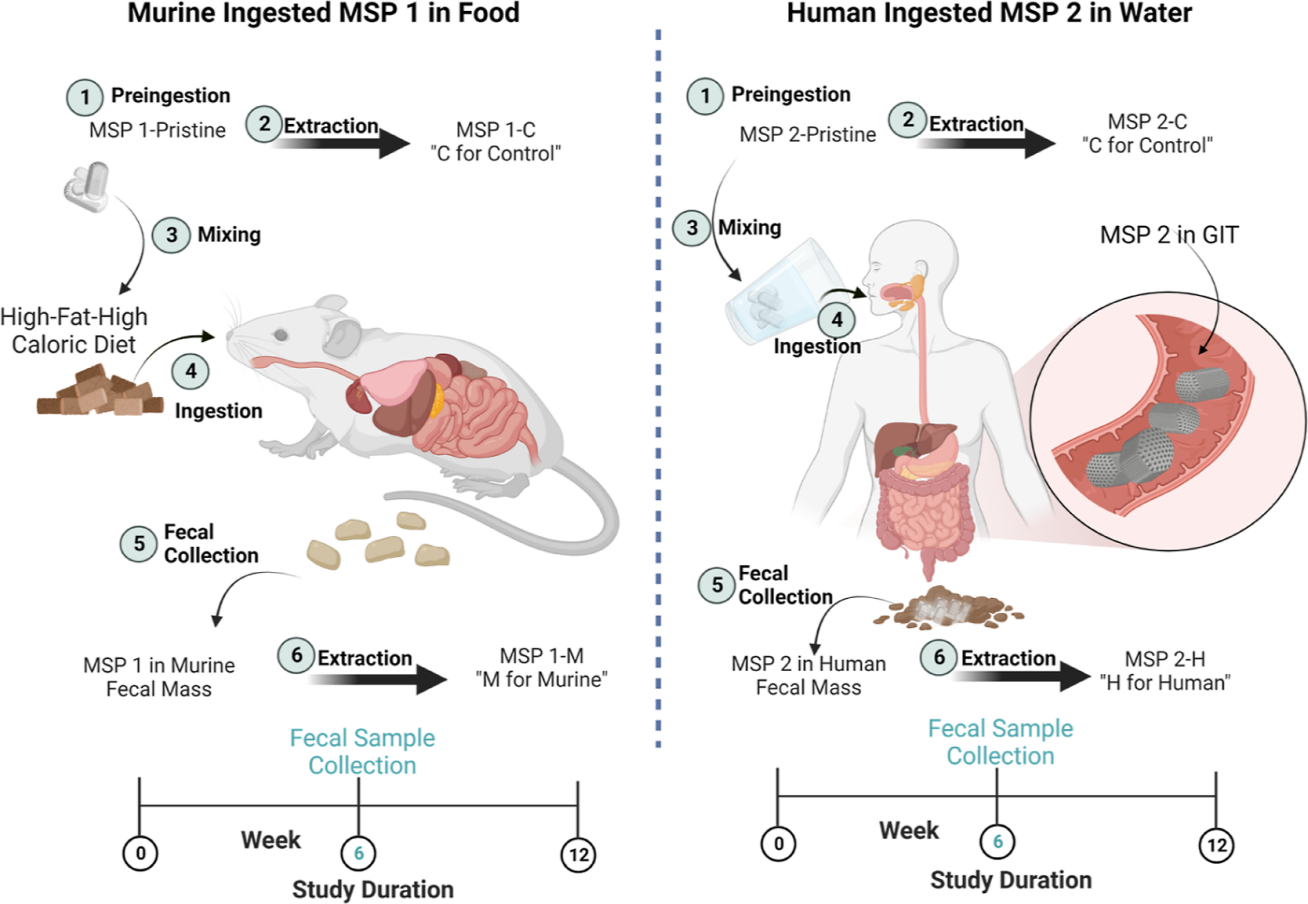
Overall Design of the Studies for the Administration of MSPs and Collection of Fecal Samples for Two Separate Batches (MSP 1—Small Batch) in the Murine Model and (MSP 2—Large Batch) in Humans

The general plan for this investigation is represented in Scheme 1, which describes the administration of the two large batches of MSPs to mice and humans, as well as the collection of samples for further extraction to perform structural and functional assessment.

#### Biosorption Assay for Porcine Pancreas α-Amylase

The adsorption of porcine pancreatic α-amylase (A6255—Sigma-Aldrich, Merck) on the MSPs was studied using a colorimetric bicinchoninic acid (BCA) (Bicinchoninic acid) assay kit (QuantiPro, Sigma-Aldrich, USA, cat no. QPBCA). Dispersions of MSPs were prepared in MQ H_2_O (milliQ—from MilliQ system of Merk, Germany) by sonicating in a batch sonicator for 30 min at a concentration level of 500 μg/mL to achieve a homogeneous dispersion. 60 μL of the dispersion was loaded in a 96-well polymerase chain reaction (PCR) plate. Porcine pancreas α-amylase (Sigma-Aldrich, cat no. A6255) suspensions were prepared from a stock of 20 mg/mL concentration in the range of 20–200 μg/mL in phosphate-buffered saline at pH 5.4 (Medicago, cat no. 1131048). 60 μL of different concentrations of pancreatic α-amylase was loaded on the same silica dispersion holding a 96-well plate. The plate was sealed and incubated at 37 °C for 3 h with vertical rotation (Harvard apparatus, cat no. 74-2302). Following incubation, the plate was centrifuged at 6200 G-force for 15 min to separate the MSPs from the supernatant. 60 μL of carefully drawn supernatant was transferred to a new flat-bottom plate (Bio-Rad, cat no. MLL9601) for measuring with BCA assay. The plate was incubated for another 60 min together with the BCA mixture in a 60 °C preheated oven. The plate was cooled, and measurements were performed at 562 nm using an absorbance reader (EnSpire, PerkinElmer, USA). Two separate 96-well plates were used, one for each batch of MSPs. In parallel, these plates were loaded with an increasing amount of α-amylase without the MSPs. Simultaneously, the absorbance values were blanked in the absence of silica and amylase. Finally, a standard curve was recorded, as shown in Figure S-1, and fitted linearly. This fitting was used to calculate the initial and final concentration of α-amylase. Successively, the quantity of α-amylase adsorbed by the silica (*Q*_e_) at equilibrium was determined as *Q*_e_ = (*C*_i_ – *C*_e_)*V*_tot_/*m* (silica). *C*_i_ is the initial concentration of α-amylase, *C*_e_ is the concentration of α-amylase at equilibrium, *V*_tot_ is the total volume, and *m* (silica) is the mass of silica. The adsorption capacity (*m*_max_), as well as the interaction constant (*K*) and the heterogeneity parameter (*n*) were obtained by a non-linear regression analysis that minimized the sum-squared deviations between the experimental data for the specific amount of adsorbed protein (*Q*_e_) as a function of the protein concentration (*C*_e_) and that given by the Hill model for adsorption.
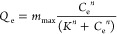
1

#### Extraction of MSPs from Fecal Mass of Mice and Humans

Treatment and analysis performed at various stages of the MSP extractions from fecal masses are reported herein. More details on the study of oral consumption of MSP 1 and MSP 2 are reported in separate communications.^14,16^ Three consecutive extractions were performed on the FD fecal samples. Extraction of MSP 1 and MSP 2 was performed from mice and human fecal samples under 20% v/v acidic conditions. Each extraction was performed under stirring and reflux conditions for around 24 h. For each extraction, different solvents were used to remove different components from the fecal samples leaving behind only the pure silica particles. FD fecal samples were poured into a glass flask, followed by different solvents (toluene/ethanol/water) and 20% v/v hydrochloric acid. A ratio of 1 g of FD fecal sample to 100 mL of solution was kept. The solvent used in the first extraction was toluene, the second extraction was performed with ethanol, and high purity water was used in the third extraction (all solvents were bought from VWR, Sweden). After each extraction procedure, the particles were filtered and washed with ethanol (4×-25 mL), followed by washing with water (2×-25 mL) and finally with acetone (3×-25 mL). Munktell grade 3 filter papers (VWR, Sweden) were used for particle separation under vacuum filtration. The extracted MSPs were poured into the round-bottom glass flask directly from the filter paper, without applying a drying procedure in between the successive extractions. At the end of the final extraction and drying, the particles were calcined at a temperature of 550 °C (in air) with 10 h of ramp time and 6 h of dwelling time. White powder obtained at the end of this fecal extraction workup procedure was labeled as “MSP 1-M” and “MSP 2-H.” In MSP 1-M, the suffix “M” stands for “murine” (extracted from the animal in vivo study),^16^ and “H” in MSP 2-H stands for “human” (from human oral ingestion study).^14^ “MSP 1-C” and “MSP 2-C” denote the control samples without ingestion but processed using the same extraction procedure as MSP 1-M and MSP 2-H.

**Scheme 2:**
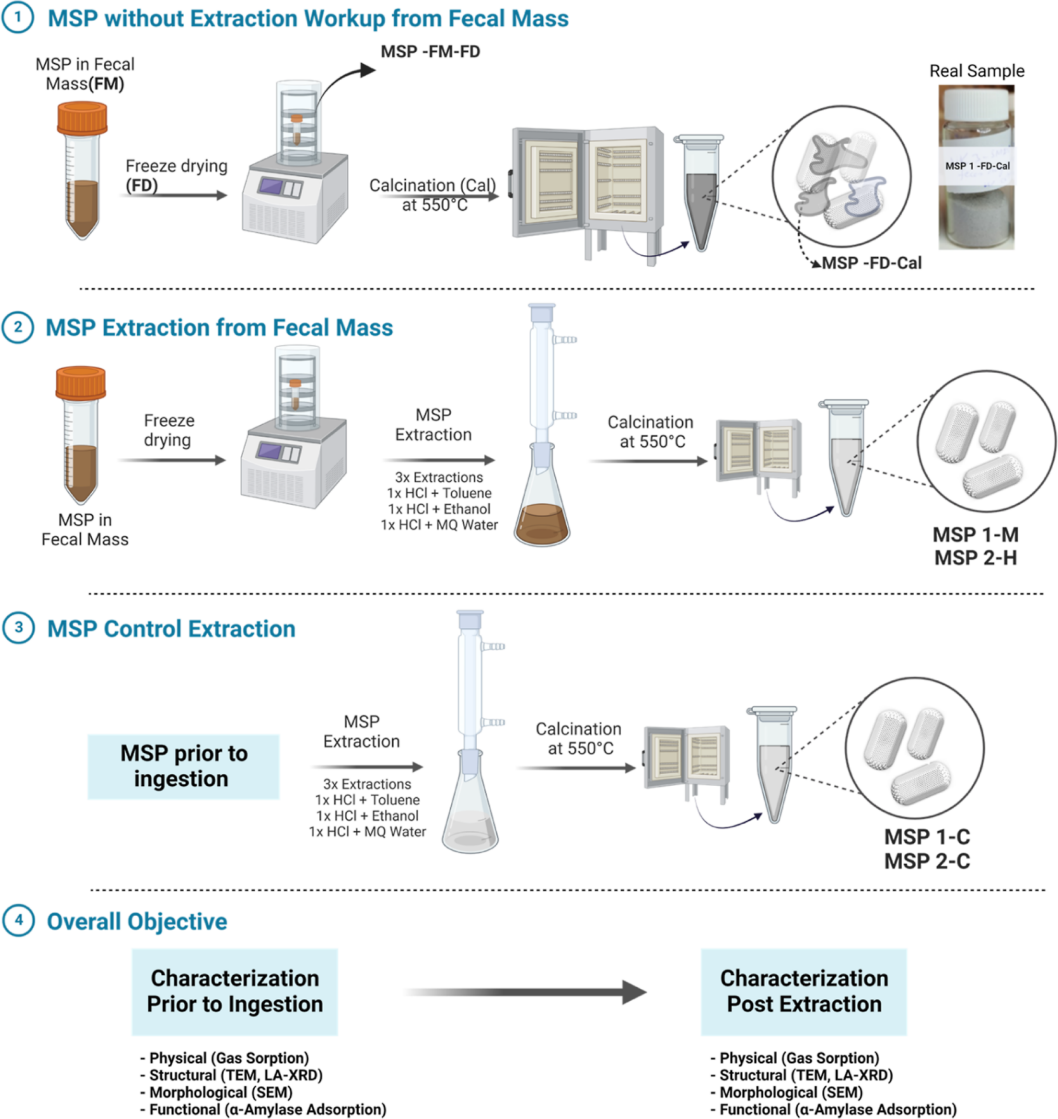
Extraction and Calcination Procedures Applied to Ordered MSPs from Mice Feces (MSP 1-M) and Those from Human Feces (MSP 2-H) MSP 1 and MSP 2 before ingestion were used as control particles and processed under the same conditions as feces samples, and these post-processed MSPs were labelled as MSP 1-C and MSP 2-C. Characterization data on pre-ingested (pristine MSP 1/2) and MSP in the fecal mass from step 1 are provided in the Supporting Information.

The overall extraction workup, calcination procedure, and sample labels are illustrated in Scheme 2. MSPs extracted from murine or human fecal samples were stored at room temperature (after steps 1 and 2 of Scheme 2), for all the characterization (as per step 3 of Scheme 2).

## Results and Discussion

### Extraction and Subsequent Calcination of MSPs from Murine and Human Fecal Mass

To study the stability of the particles of SBA-15 in the GIT environment of mice and humans, we derived a method to extract particles from the mass from oral ingestion studies in murine and human models.^14,16^ White powders were obtained, and the method is illustrated in Scheme 2, step 2. The fecal mass was FD, extracted by acidified solvents, and subsequently calcined. In parallel, we studied control samples (Scheme 2, step 3). Without the extraction step, it was not possible to obtain the particles in their purest form (as illustrated in Scheme 2, step 1). The purpose of developing the procedure was to extract these particles from fecal mass and examine their physical, morphological, structural, and functional characteristics. It can be noted that oral administration of MSPs in humans has been shown well tolerable and safe at doses of 9 g/day.^14,15^

### Pore Size Analysis of the MSPs

**Figure 1:**
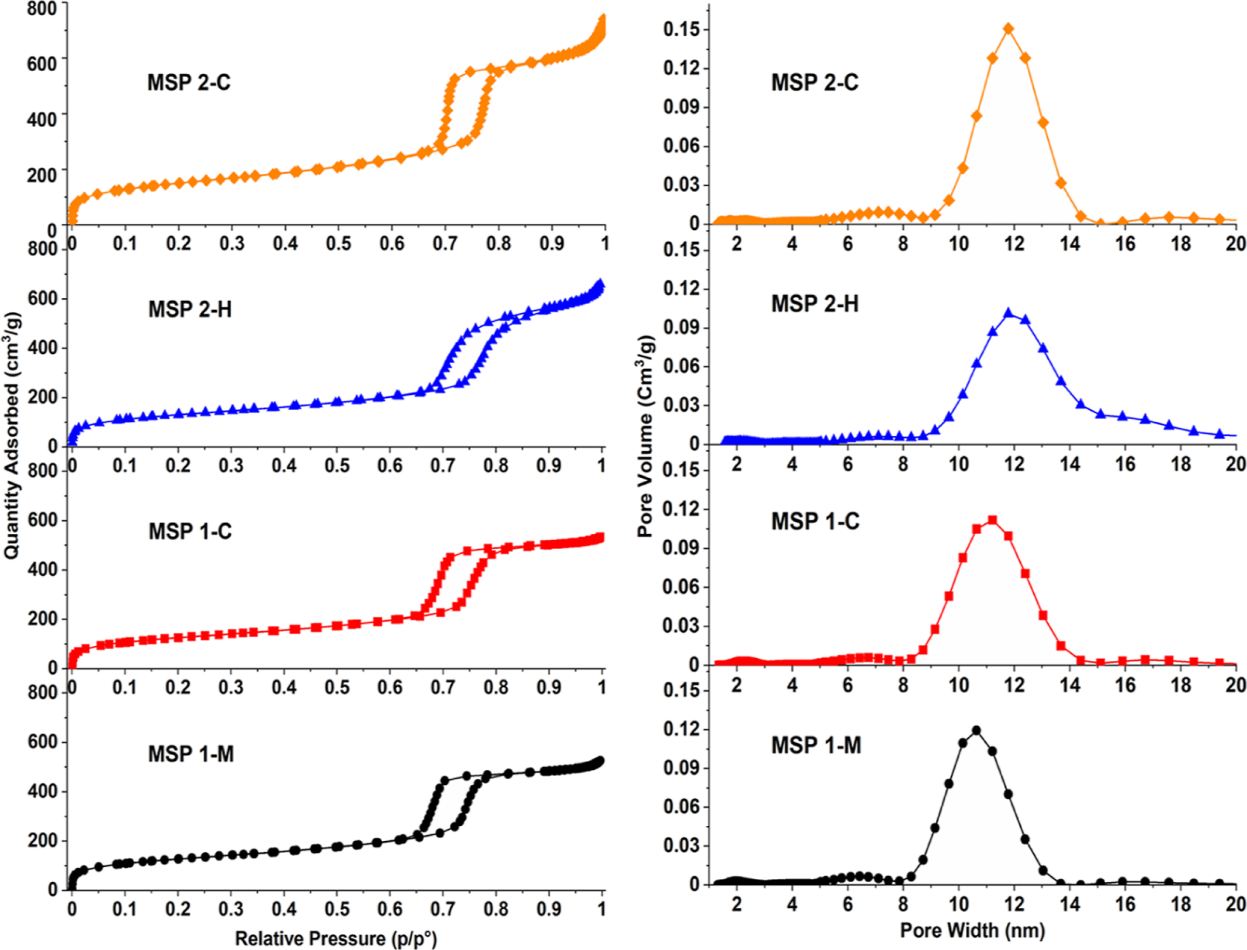
N_2_ gas adsorption isotherms (left) and respective PSD (right) for MSPs recovered from mice feces with a joint extraction and calcination procedure (MSP 1-M) and the corresponding control (MSP 1-C). MSP 2-H was recovered from human fecal mass by a joint extraction and calcination procedure and MSP 2-C is the corresponding control. Comparison with Scheme 2 (steps 2 and 3).

MSPs are commonly analyzed by N_2_ adsorption.^30^ Such analyses have allowed to study structures and validate density functional theory (DFT) models.^30^ The N_2_ adsorption–desorption isotherms and pore size distributions (PSDs) of the SBA-15-type particles of this study are presented in Figure 1. The particles that had passed through the GITs of humans or mice had similar PSDs to those of the control samples.^31^ The mesopores were not significantly affected by the GIT passage or the acid extraction. We ascribe the stability to the chemistry involved, and the high degree of siloxane-rich bonding after the calcination performed at 550 °C.^32,33^ The joint extraction and calcination procedure did, however, expand the pores and broadened the PSD somewhat [full-width half maxima (fwhm) in Table 1, PSD in Figure 1] compared to the Pristine MSPs. Also, MSP 2-C had slightly narrower PSD than MSP 1-C, and the controls had slightly different pore sizes (11.0 ± 0.1 nm for MSP 1-C and 11.9 ± 0.1 nm for MSP 2-C).

**Table 1:** Material Properties as Measured by N_2_ Adsorption Isotherms and Low-Angle X-ray Diffraction for MSP 1-Pristine, MSP 2-Pristine, Extracted and Calcined Samples MSP 1-M (from Feces of Mice) and MSP 2-H (from Feces of Humans), and Controls, Extracted and Calcined MSP 1-C and MSP 2-C^a^

sample	BET surface area (m^2^/g)	total pore volume (cm^3^/g)	micropore surface area (m^2^/g)	pore size (DFT) (nm)	fwhm* (nm)	*d*_100_ (nm)	*a*_o_ (nm)
MSP 1-Pristine	615 ± 6	0.82 ± 0.01	207 ± 6	9.5 ± 0.05	1.5 ± 0.01	9.6	11.1
MSP 1-C	429 ± 25	0.80 ± 0.02	95 ± 18	11.0 ± 0.12	2.8 ± 0.07	9.6	11.1
MSP 1-M	437 ± 25	0.80 ± 0.01	95 ± 12	10.7 ± 0.04	2.5 ± 0.04	9.7	11.2
MSP 2-Pristine	857 ± 66	1.15 ± 0.05	370 ± 48	10.1 ± 0.10	1.5 ± 0.03	10.3	11.9
MSP 2-C	553 ± 23	1.11 ± 0.04	131 ± 11	11.9 ± 0.05	2.4 ± 0.00	10.1	11.7
MSP 2-H	462 ± 12	1.04 ± 0.02	105 ± 12	12.1 ± 0.01	3.3 ± 0.02	10.1	11.7

a Values are the mean of triplicated measurement ± standard deviation. Sign (*) is the full-width half maxima.

Calcined SBA-15 particles are known to have both meso- and microporosity,^34,35^ and values for the particles of this study are presented in Table 1. The micropore surface areas for particles passing through the GITs were similar to that of the control samples. The slightly higher value for MSP 2-C compared to MSP 2-H was assigned to a minor inclusion (about 10%) of an inert in MSP 2-H. As can be seen in Table 1, the Brunauer–Emmett–Teller (BET) surface area, pore volume, and micropore surface area for all worked-up samples were lower, and the pore sizes were larger, as compared to their pristine counterparts. We conclude that the acid treatment reduced especially the micropore surface area. Possibly, this lowering could be attributed to a surface cleaning effect during the extraction workup procedure. Similar effects have been observed by others during reflux in water.^36−38^

We also studied MSPs recovered without the extraction workup procedure. In this case, we used freeze drying and tested the effect of a subsequent calcination procedure (Scheme S-1). FD samples that were not calcined (MSP 1-FM-FD and MSP 2-FM-FD) had smaller average pore sizes (Figures S-3 and S-4, middle-green) than those subjected to calcination (MSP 1-FM-Cal and MSP 2-FM-Cal). Even without the calcination, the MSPs leaving the GIT had empty pores up to about 8 nm. After calcination, the pores had a larger average size of 10–12 nm. Based on our previous findings and obvious geometric constraints, we expect that biomolecules in the GIT such as α-amylase and lipase do not enter the smallest of the pores, whereas when the MSPs contain large enough mesopores, they adsorb on the walls of the large pores during passage through the GIT.^2^ Nonetheless, these sufficiently large mesopores are emptied from molecular occupancy upon calcination. The presence of uncalcined fecal mass was apparent from the gas sorption analysis of the MSPs not subjected to the extraction workup procedure. However, the N_2_ adsorption analysis of MSP 1/MSP 2-FD-Cal showed that the pores structures largely remained unaffected after the GIT passage.

### Analysis of SEM Images of the MSPs

**Figure 2:**
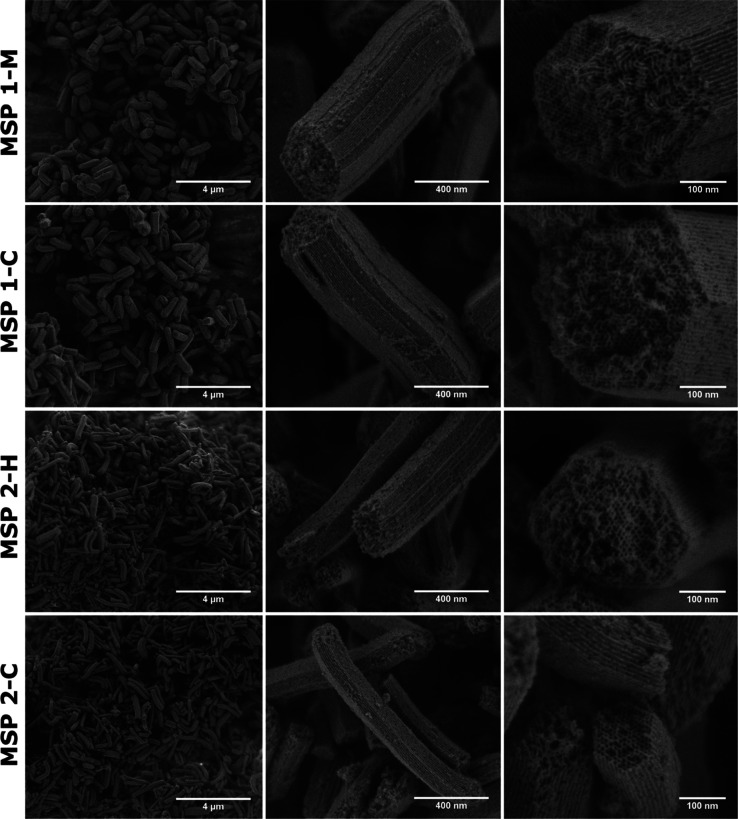
SEM images at different magnifications of MSP 1 and MSP 2 processed following Scheme 2, after a joint extraction and calcination procedure of fecal samples of murine (M) and human (H) along with images of respective control (C) samples.

Particle size analysis before the ingestion and after recovery from fecal mass showed similar lengths and widths of the particles (see Figure S-5). The particles were elongated, facetted, and contained mesopores that run in parallel with the long axes of the particles. This can be seen from the SEM images in Figure 2. Generally, the primary micron-sized particles were agglomerated. Overall, the high-resolution SEM images showed that the SBA-15 particles were faceted rods. The average length of the MSP 1 rods was almost 1 μm and slightly longer for MSP 2. The extracted particles (controls and those that had passed the GITs) had fine lateral pores running perpendicular to the main mesopores. These lateral pores connected the main mesopores. High-resolution surface imaging showed that these lateral pores had a range of sizes, both in the micro- and mesopore domains. These findings connect well to studies of others.^35,39−43^ The SEM analysis revealed no discernible morphological difference between the particles before and after ingestion and a joint extraction and calcination procedure. This similarity supports our hypothesis that the MSPs pass through murine and human GIT without being altered morphologically. For MSP 2-Pristine, some broken particles were observed (Figures S-2 and S-5) and are related to the large-scale manufacturing process. This batch was dried on the filtration system before the calcination step, and it seems that some particles have been broken mechanically during this drying. At this stage, the uncalcined precursors of the MSPs (the mesostructured silica-polymer composites) were malleable and possibly susceptible to breakage under mechanical movement.

### Analysis of TEM Images of the MSPs

**Figure 3:**
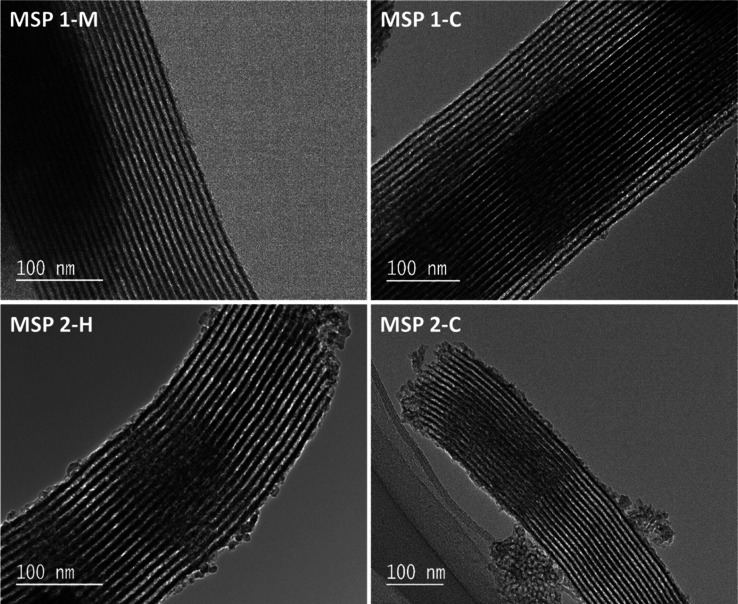
TEM images of samples, after applying a joint extraction and workup procedure from fecal samples of murine (MSP 1-M) and its control sample (MSP 1-C), alongside a sample after human fecal extraction and calcination (MSP 2-H), and control sample processed in parallel (MSP 2-C).

**Figure 4:**
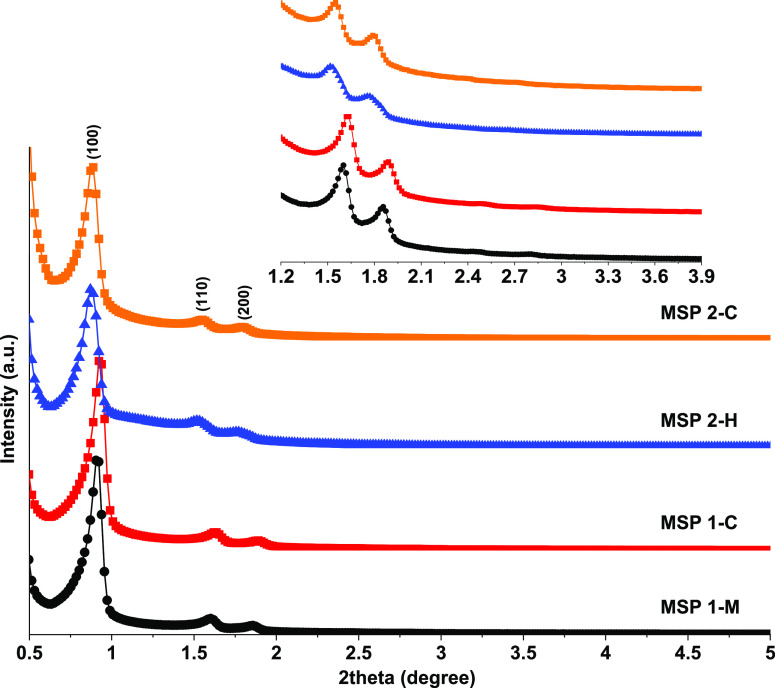
Small-angle X-ray diffraction curves for the samples extracted from fecal mass (MSP 1-M and MSP 2-H) and corresponding control samples (MSP 1-C and MSP 2-C). The inset shows the enlarged part of peaks indexed to (110) and (200) diffractions for visualization purposes.

**Figure 5:**
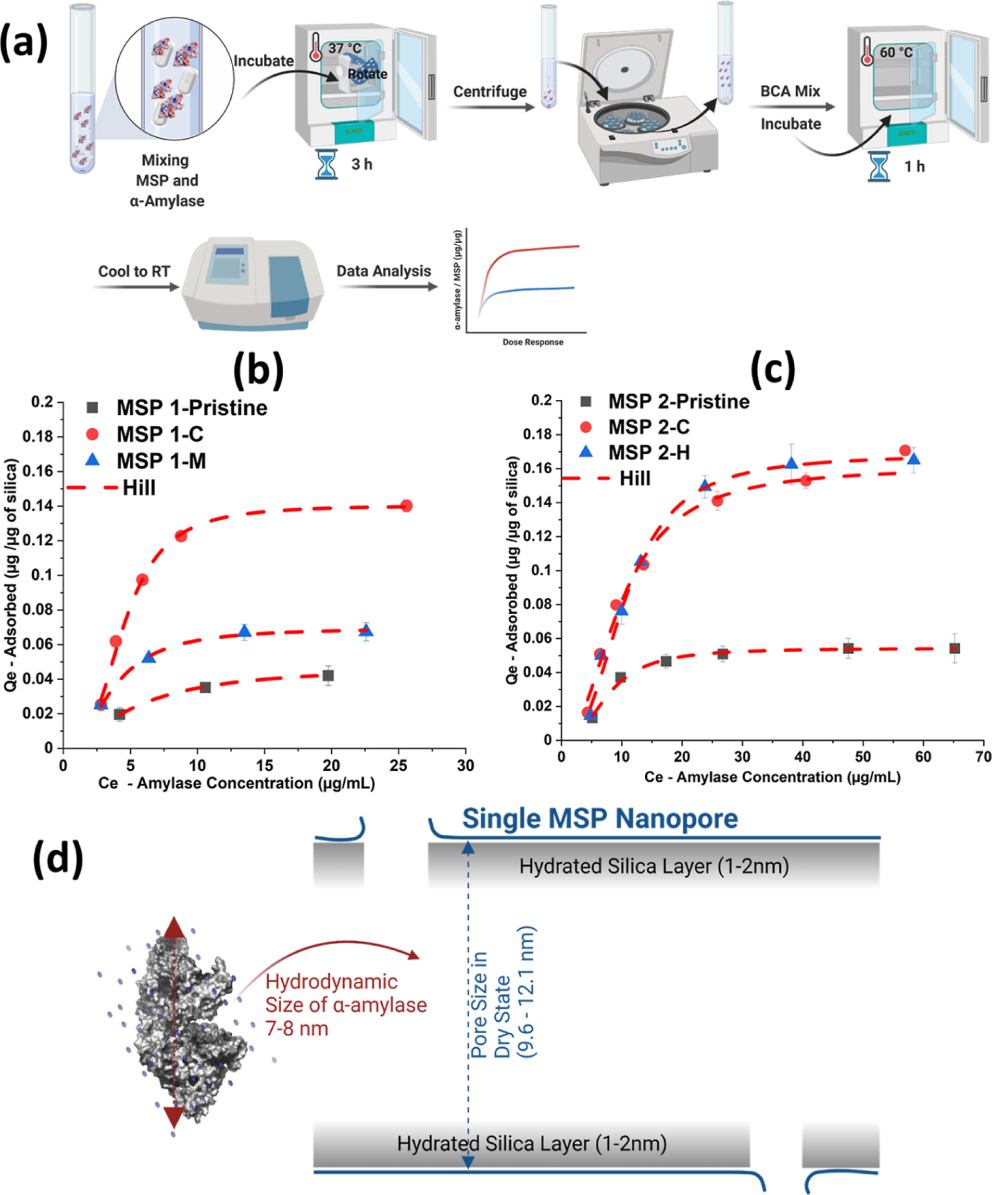
Scheme in (a) shows functional testing of the MSPs. (b,c) Mass fraction of porcine α-amylase adsorbed on the MSPs used in a murine model (b) and administered to human model (c) for before (pristine), extracted, and control samples. Fitting data are presented in Table S-1. Standard curves are shown in Figure S-1. Results are presented as mean ± SD. (d) Illustration of the nano-bio size interaction of α-amylase at a single molecule and pore level.

TEM was used to study the structural characteristic of MSPs at the individual particle level, and mesopores are seen for all samples before and after passage through the GIT (Figure S-6 for Pristine and Figure 3). The images were taken with the mesopores positioned perpendicular to the electron beam, whereby a side view of pores was observed, resulting in a stripe pattern. The primary mesopores are cylindrical with a constant diameter and a length equal to the particle length. These cylindrical pores are going through the particles, as can be seen in Figures 3 and S-6. The mesopores could have been preserved by a protective action from the high-quality samples, a short residence time, or uptake of size-specific enzymes such as α-amylase and lipases, as shown in our previous reports.^2^ The protective role of digestive enzymes can be attributed to restricting access to surface eroding attacks of GIT fluids on the silica.^44^ The calcination and hydrothermal procedures adopted in this work for the synthesis of the MSPs are also providing extra stability. It is worth mentioning here that no degradation was observed for the extracted silica particles or in the controls. It was the case even when the extraction was performed under highly acidic conditions for 72 h post-GIT excretion.

### Structural Analysis of the MSPs Using X-ray Diffraction

In an earlier study of extracted MSPs from a simulated body fluid, damaged pore structures were observed after 3 days of incubation.^19^ However, no such pore structure deterioration was observed by the GIT passage and subsequent acid extraction and calcination of the MSPs in this study. The ordered pores of the MSPs led to X-ray diffraction at small angles, as can be seen in Figure 4. No significant differences in the intensity and position of diffraction peaks were observed between control samples and those passing through the GITs. The diffraction peaks were indexed as (100), (110), and (200) diffractions of 2D-hexagonal mesoporous ordering.^5,30,45^ The extraction and calcination procedure had a minor impact on both the diffractograms of the MSPs of the control and post-GIT excreted types. When compared to the diffractograms of their pristine counterparts, the intensities of the diffractograms of the controls, MSP 1-M, and MSP 2-H were slightly reduced. MSP 1-Pristine, MSP 1-M, and MSP 1-C had *d*(100) spacings between 9.6 and 9.7 nm and cell parameters (*a*_0_) of 11.1–11.2 nm, as reported in Table 1. The cell parameter was slightly larger for MSP 2-Pristine, MSP 2-H, and MSP 2-C at 11.7–11.8 nm. This difference between the batches was consistent with N_2_ adsorption analysis. The diffractograms for MSP 1/2-Pristine are presented in Figure S-7.

We have previously shown that the MSPs studied here led to significant attenuation in weight gain in mice and reduction in long-term plasma glucose (HbA1c) levels in humans.^2,14−16,46^ They were ingested with or before food intake. In line with this effect, we hypothesized that amylase and lipase were adsorbed in the particles and further interactions with substrates were hindered.^2^ As the internal specific surface area exceeds significantly the external one, surface interactions inside the pores have an auxiliary effect.^46−49^ Physical separation and molecular retention in the pores protect particles from gastrointestinal (GI) fluids and physio-chemical disintegration.^50^ It can be noted that particle–protein complexes are known for porous silica^51,52^ and silica nanoparticles.^53^ This “protein corona” can control the biological fate of the silica particles and ultimately modulate the biological response, biodistribution, and biostability.^28^ Nanoparticles could accumulate over and across the GIT wall and have undesired effects. This is precisely why larger and micron-sized particles of the MSPs were used in this study. However, a breakdown of the MSPs could result in some formation of nanoparticles. This is why we investigated the structure of MSP after passage through the GIT in this study, aiming to confirm that significant degradation of the mesoporous particles did not happen. If minimal degradation were to occur, this would result in the formation of small amounts of orthosilicic acid through partial dissolution of the silica. However, absorption of small amounts of orthosilicic acid is not a safety concern because several studies have investigated the absorption of orthosilicic acid and have shown that it is not accumulated in any tissue and excreted mainly via the kidneys.^54^ Furthermore, thermogravimetric analysis of the feces collected in the mice study showed that the difference in inorganic material between the HFHC control and the MSP-1 supplemented groups roughly corresponded to the ingested MSP-1 dose, confirming that the MSP-1 passed through the GI system without absorption.^16^

When using MSPs as delivery vehicles for the oral administration of drugs, the drug payload is adsorbed on the internal surfaces of the pores or volumetrically as pore filling. Once the pores have been emptied of the drug payload, the MSPs could interact and adsorb also other molecules. We have previously shown that MSP adsorb digestive enzymes (α-amylase and lipase) from GI juices.^2^ It is possible that MSP when used for oral drug delivery adsorb digestive enzymes after release of their payload if the pores are sufficiently large.

### Biosorption of MSPs

To further assess to what extent proteins from the GI fluid are adsorbed on the MSPs, we constructed a model system. The adsorption of porcine pancreatic α-amylase was studied on the particles of mesostructured silica. α-Amylase was selected because of its action of splitting amylose into maltose units, its importance in the digestive system, and its potential links to diabetes and pre-diabetes.^55^ In addition, we have previously studied the adsorption of α-amylase by MSP, its effect on starch digestion, and the potential applications in obesity and diabetes prevention.^2^ The adsorption isotherms are presented in Figure 5 and described in a Hill model. It included the adsorption capacity (*m*_max_), interaction strength (*K*), and Hill coefficient relating to the heterogeneity of the heat of adsorption (*n*).^5,56,57^ For the adsorption capacity, three of the extracted samples (the controls and MSP 2-H) had high values [14–17% (w/w)]. This capacity corresponded to a filling degree of about 35–40% for α-amylase in the pores. The pristine samples and MSP 1-M had lower filling degrees (12–18%). These data are in the same range compared to previously reported loading capacity for other porous silica particles as the loading capacity of α-amylase in mesostructured cellular foam (MCF) of silica with 24 nm pores was 160 mg/g,^58^ as compared to 170 mg/g in MSP 2-H/C samples. However, the starting incubation dosage of enzyme for MCF porous particles was high, at 3 mg/mL, with having little physiological relevance. A nano-bio molecular size fit analysis was performed for the MSPs, and it is presented in Figure S-8. It showed a transition from a lower adsorption capacity toward a higher one. The critical pore size was 10.8 nm. Above this value, the adsorption capacity was high. This critical size was consistent with the sum of the hydrodynamic size of α-amylase (7–8 nm) and the size of the two hydration layers, as illustrated in Figure 5d. The hydrated layer on silica is well documented.^59^ For the interaction strengths of the adsorption (the *K* values), one batch had small values (MSP 1) and an apparent smaller level of silica–amylase interactions. This aspect was not further studied. For the heterogeneity parameters, values were similar and typically above two (the values for *m*_max_, *K*, and *n* are presented in Table S-1.).

In this study, we also show that the adsorptive capacity to α-amylase is enhanced by the joint extraction and calcination procedure, that the MSPs survive the passage through the GIT, and that they display at least as high adsorptive capacity as the control samples. This enhancement can be of importance in further clinical studies of MSPs inducing weight loss and as a therapeutic agent in reducing hyperglycemia. It can be noted that when MSP 1-Pristine was fed in food to mice, significant weight loss was observed in a preclinical setting. It has been linked to a reduction of the food intake efficiency.^16,46^

## Conclusions

We have shown for the first time that MSPs retain their physical, structural, morphological, and functional characteristics after passing through the digestive tracts of mice and humans. We analyzed the MSPs in the feces of humans and mice after an extraction and calcination work-up procedure. To reach this conclusion, we developed a method to extract particles from fecal mass and performed studies with N_2_ adsorption/desorption, TEM, SEM, XRD, and adsorption of porcine α-amylase. The extraction involved acid in different solvents. This protocol enabled a novel examination of the biostability of the MSPs. The silica particles retained the meso/microporosity, structural order, and particle morphology. A small amount of microporosity was lost during the extraction procedure. The minor discrepancies between the two batches of particles were ascribed to the scale-dependent conditions of the synthesis and calcination. The adsorption level of α-amylase was enhanced for the extracted porous silica particles and related to the pore dimension, hydrodynamic size of the enzyme, and the hydration layers on the internal silica pores.

The pores of MSPs passing through the GIT were also found to be empty below 8 nm in size, as determined by examination of the MSPs without extraction work up. In contrast, bigger pores were found to be occupied by organic matter which we attribute to the adsorption of GIT biomolecules such as α-amylase, in line with our previous findings.^2^

It was notable that the acid-extracted and calcined MSPs had a higher capacity for α-amylase adsorption (2–3 times) than pristine MSPs. Higher *K* values (in the Hill model) were observed for MSP 2 than that for MSP 1 which had slightly smaller pores. It is of interest to direct further studies toward determining the role of the mesoporous structure in greater depth. Such studies could also focus on studying the details of the molecular interactions in relation to biomolecules adsorption and desorption in the MSPs and how that relates to biological function. Further studies of the adsorption capacity and adsorption kinetics of digestive enzymes should be taken into account for future practical usage of MSPs in drug delivery and therapeutic applications.

The proven robustness of the particles passing through the digestive tract, including retaining their function, is of high importance for the use of mesostructured silica in food additives, therapeutic uses, and oral delivery vehicles, including a number of future oral clinical applications. The biostability in murine and human GITs supports a high safety window of these particles since the concentration of degradation products of the particles in the intestine would be non-existent or very low.

## Supporting Information Available

Material characterization techniques, standard curve for α-amylase concentration measurement, characterization data (SEM, TEM, LAXRD, and porosity) on pre-ingested (pristine MSP 1/2), N_2_ gas adsorption measurement for MSP 1/2 in the fecal mass, particle size analysis of MSP 2-Pristine and after GIT passage, size analysis of α-amylase in MSP, and fitting data for adsorption capacity and interaction of α-amylase in MSPs (PDF)
