# Supplementary material for: Mesoporous Silica Particles Retain Their Structure and Function while Passing through the Gastrointestinal Tracts of Mice and Humans
Source: ACS Appl Mater Interfaces. 2023 Feb 2;15(7):9542–53. doi: 10.1021/acsami.2c16710 (PMC9951217; doi:10.1021/acsami.2c16710)
Supplement: Supplementary file 1 — am2c16710_si_001.pdf [file am2c16710_si_001.pdf]

Supporting Information for

Mesoporous Silica Particles Retain their Structure and Function While Passing through the Gastrointestinal Tracts of Mice and Humans

Muhammad Naeem Iqbal[a,b], Ghislaine Robert-Nicoud[a,b], Marina Ciurans-Oset[c], Farid Akhtar[c], Niklas Hedin[a*], Tore Bengtsson[b,d*]

[a] Department of Materials and Environmental Chemistry, Stockholm University, Stockholm, SE-106 91, Sweden

[b] Sigrid Therapeutics AB, Stockholm, Stockholm, 113 29, Sweden

[c] Division of Materials Science, Department of Engineering Sciences and Mathematics, Luleå University of Technology, Luleå, SE-971 87, Sweden

[d] Department of Molecular Biosciences, The Wenner-Gren institute, Stockholm University, Stockholm, SE-106 91, Sweden

[*] Corresponding Authors

[niklas.hedin@mmk.su.se; tore.bengtsson@su.se]

Number of Pages: 12

Number of Figures: 8

Number of Tables: 1

All the results are plotted using Origin 2020 (Origin Lab, USA). All the illustration were prepared in biorender.com

Materials Characterization

Nitrogen sorption analysis

N_2 sorption analysis was performed with a Tristar II instrument (Micromeritics, USA) at the temperature of liquid N_2. The MSP 1 & MSP 2 samples (before ingestion and worked up samples after digestion) were subjected to dynamic vacuum conditions and a temperature of 150°C for at least 19 hours before being studied with N_2 gas sorption. MSP samples analyzed directly in the fecal mass (FM) were degassed at a lower temperature of 80°C. The specific surface area was determined in the BET model using adsorption data in the relative pressure regime of 0.05–0.20. The total pore volume was determined by single point adsorption at a relative pressure close to unity. The micropore surface area and pore volume were determined by the t-plot method within the thickness range of 0.5–0.8 nm. Density functional theory (DFT) was applied to the N_2 adsorption data using a cylindrical pore model for pore size distribution (PSD) estimation, [1] and a regularization procedure was used to avoid overinterpretation of the data, using a program from Micromeritics. All the measurements were triplicated and presented as average with errors as standard deviation.

Low-angle X-ray diffraction (XRD)

Low-angle XRD was performed on PANalytical powder diffractometer (PANalytical, Germany) operated at 45 kV and 40 mA, with 0.02° step size and equipped with Cu Kα radiation source in a transmission mode. Samples were prepared on Mylar C® film (Mylar C® film was 6 μm thick), with the incident beam perpendicular to the sample surface. Samples were sandwiched as a thin layer between two Mylar C® films. The unit cell (**a_0**) parameter was calculated using (2/√3) d_{100} from the d_{100} spacing, assuming a 2D hexagonal pore geometry.

Scanning electron microscopy (SEM)

Morphological investigations of the MSP samples before and after passing through the GIT were performed with SEM. Images were recorded with a Magellan 400 Extreme-High Resolution SEM (FEI Company, Eindhoven, Netherlands) microscope using a through-the-lens (TLD) detector. It is equipped with Schottky emitter and a monochromator for getting a tightly focused beam. The microscope was operated at an accelerating voltage of 250 V, stage bias of 2.75 kV and probe current of 3.1 pA. The working distance was altered between 2 and 3 millimeters. Samples were prepared by dispersing a small amount of particles in dichloromethane by ultrasonication for 5 minutes, depositing one drop of the dispersion on a pre-ground aluminum substrate and letting to dry before loading in the microscope. Images were taken on non-coated samples.

Transmission electron microscope (TEM)

Transmission electron microscope analysis was performed with a JEM-2100F (JEOL, Japan) microscope, equipped with LAB6 as electron-source filament, for visualizing the porous network of the MSP 1 & MSP 2 samples before and after being passed

through the GIT. It was operated at 200 kV and imaged using a bottom mounted CCD (Gatan SC1000 ORIUS) camera. Samples for TEM imaging examination were prepared by gently crushing the particles in a dry state and sonicated in pure ethanol for 10 min. A drop of this particle dispersion was then transferred to a carbon-coated copper grid.

Biosorption analysis using α-amylase from porcine pancreas

The protein adsorption experiments used α-amylase from porcine pancreas (A6255 – Sigma Aldrich, Merck). The amount of α-amylase was measured using a bicinchoninic acid (BCA) assay kit (QuantiPro, Sigma-Aldrich, USA, cat no. QPBCA). Measurements of absorbances were performed using a multimode plate reader at a wavelength of 562 nm (EnSpire, Perkin Elmer, USA).

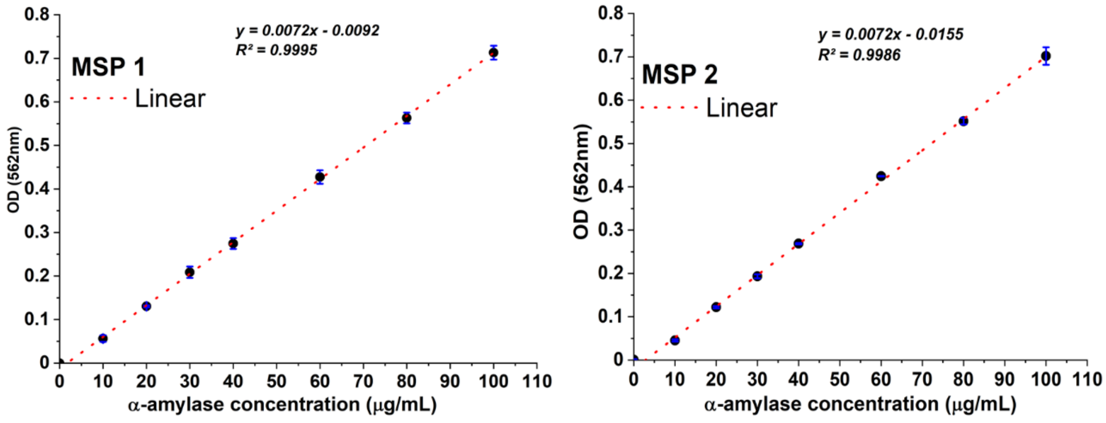

Figure S-1: Standard curve for porcine pancreatic alpha-amylase measured at 562 nm OD values, in turn used for calculating the amount of adsorbed α-amylase in MSP, shown in adsorption plots in Figure 5. Dotted lines are linear fits. Mean of triplicated measurement is plotted with standard deviation presented in error bars.

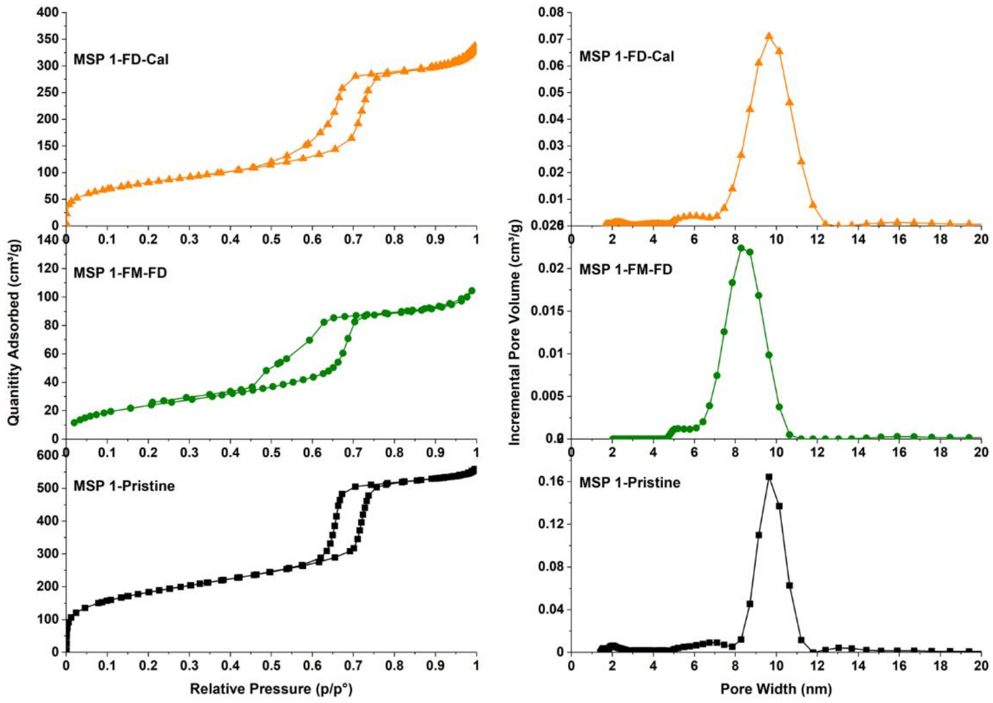

Figure S-2: N_2 gas adsorption measurement of the samples obtained using the method described in Scheme 2, step 1, plotted together with MSP 1-Pristine. PSD on the right and isotherm on the left.

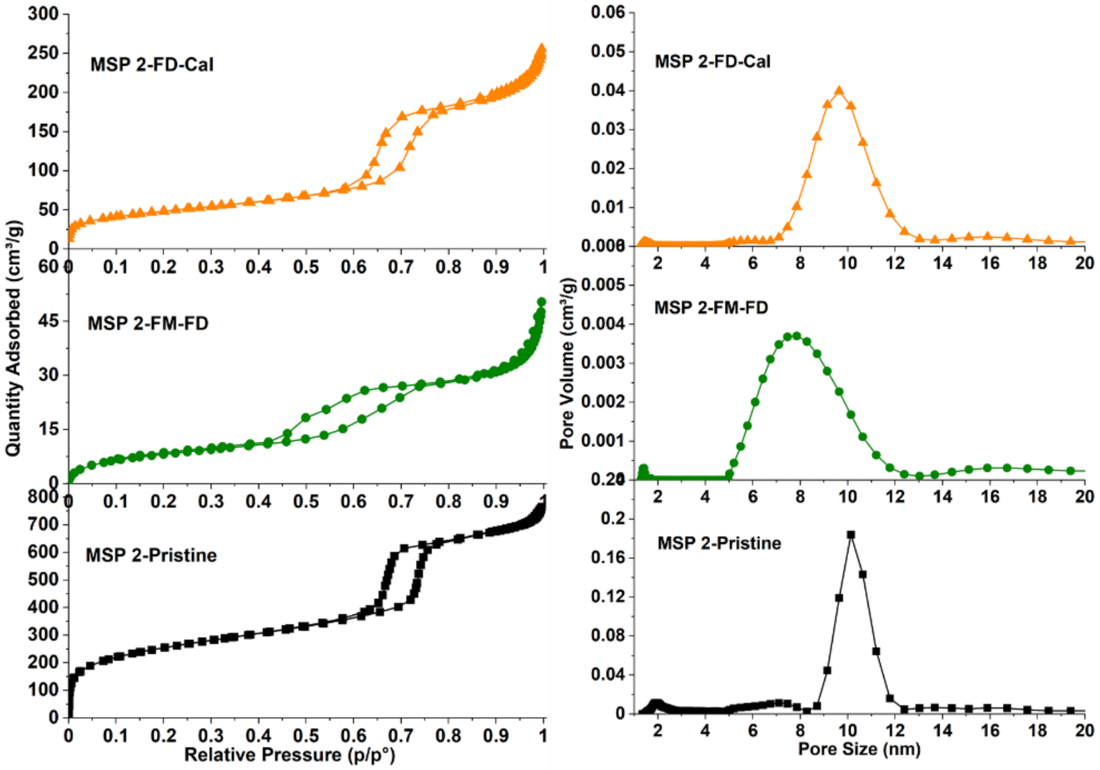

Figure S-3: N_2 gas adsorption measurement of the samples obtained using the method described in Scheme 2, step 1, plotted together with MSP 2 Pristine. PSD on the right and isotherm on the left.

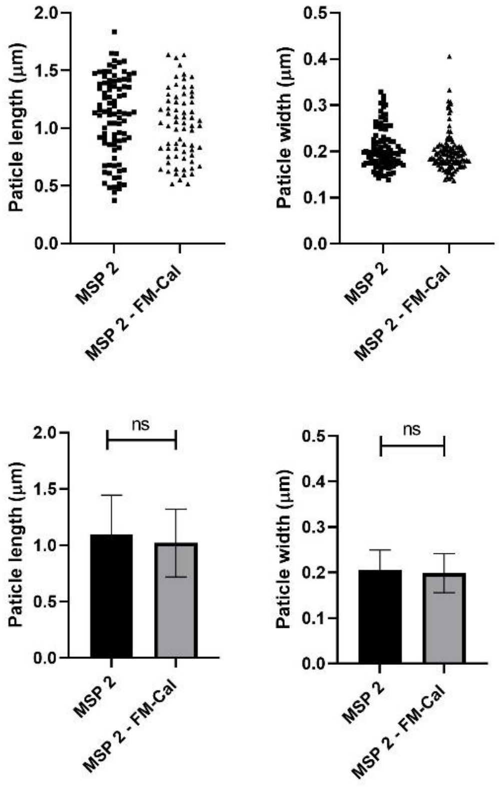

Figure S-4: Particle size analysis of MSP 2 before ingestion and in fecal mass after calcination (MSP 2-FM-Cal) measured from SEM images using ImageJ (Fiji)[2], “ns” stands for no significance, analyzed with unpaired t-test. A minimum of 50 particles are measured for length and 100 for width.

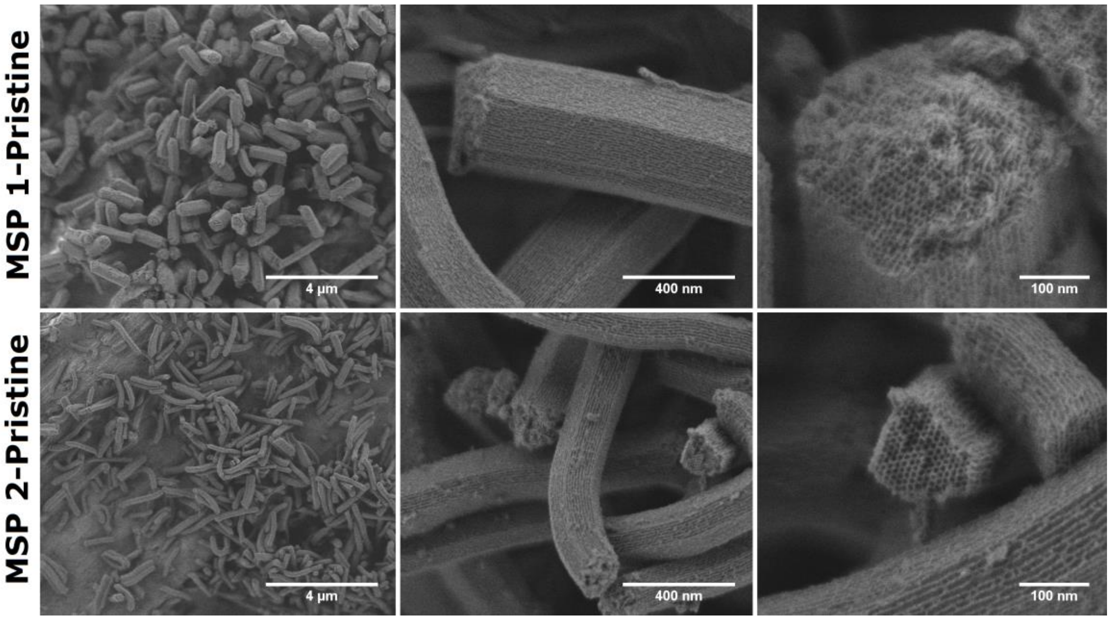

Figure S-5: SEM images of MSP 1 and MSP 2 in pristine form that is at pre-ingested stage.

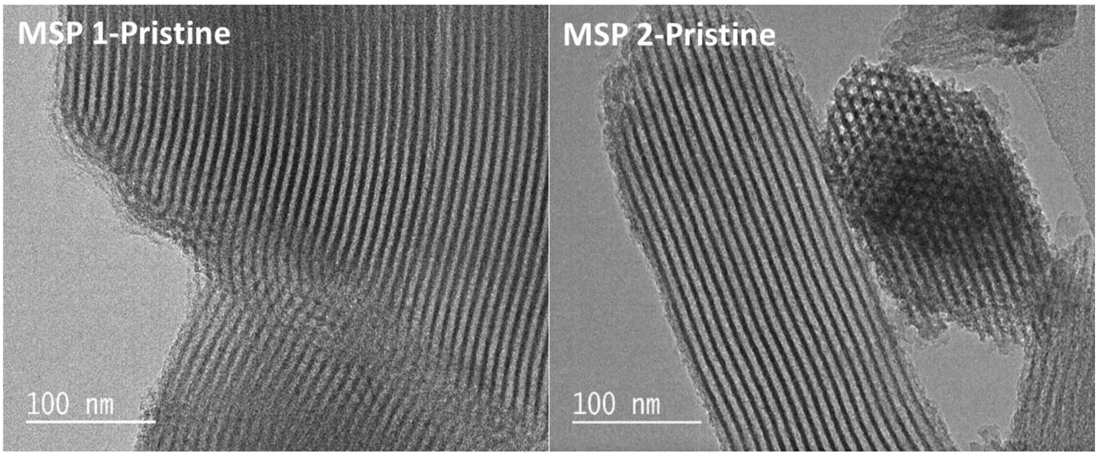

Figure S-6: TEM images of pristine MSP 1 and MSP 2.

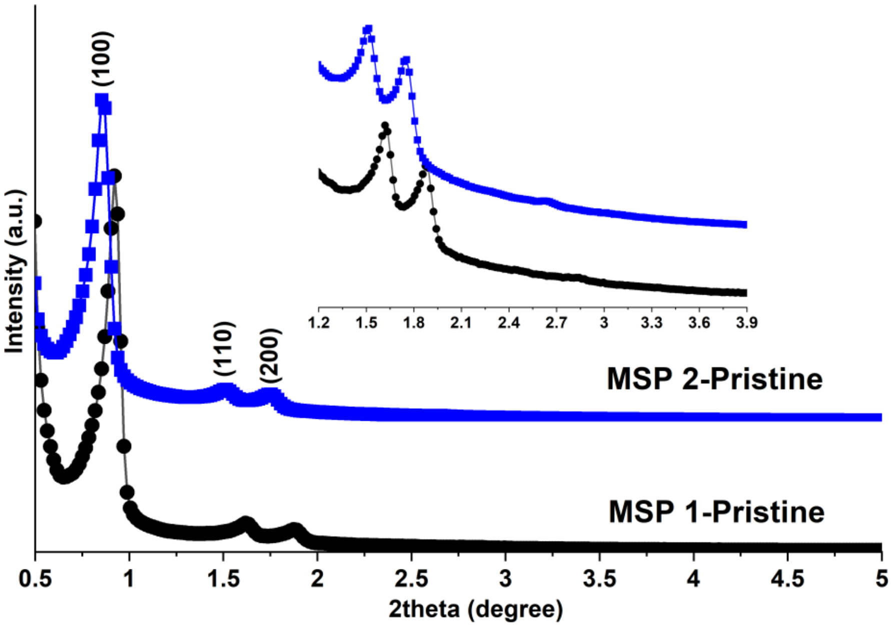

Figure S-7: Low angle XRD of pre-ingested MSP 1 and MSP 2.

Table S-1: Fitting data from MSP 1 and MSP 2, pristine, extracted after GIT passage and extracted controls, after porcine pancreatic alpha-amylase sorption.

**Plot Name**	**MSP 1-Pristine***	**MSP 1 – C**	**MSP 1 – M**	**MSP 2-Pristine**	**MSP 2-C**	**MSP 2-H**
**m_{max} (µg/µg)**	0.0475 ± 0	0.140 ± 0.003	0.070 ± 0.002	0.054 ± 8.399E-4	0.161 ± 0.008	0.168 ± 0.003
**K (µg/mL)**	5.296 ± 0	4.474 ± 0.138	3.720 ± 0.316	7.781 ± 0.378	9.830 ± 0.906	10.653 ± 0.322
**n**	1.593 ± 0	2.992 ± 0.236	2.170± 0.426	2.441 ± 0.253	2.129 ± 0.337	2.538 ± 0.229
**Reduced Chi-Sqr**	0	2.351 E-08	1.630 E-08	8.136 E-09	1.077 E-07	7.992 E-08
**R-Square (COD)**	0.999	0.998	0.992	0.994	0.991	0.997
**Adj. R-Square**	0	0.995	0.976	0.989	0.986	0.995

*Only three point are used for fitting, hence no error bars are provided.

Error values here are more qualitative, primarily presenting a goodness of fitted parameters.

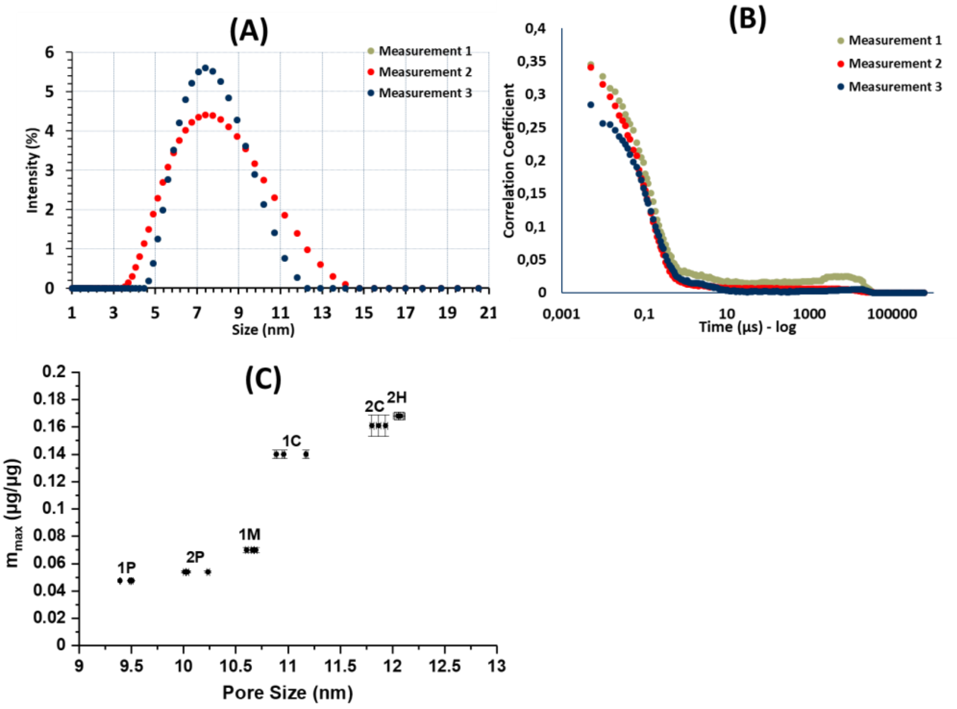

Figure S-8: A size match analysis for α-amylase and pore size of MSP; (A-B) hydrodynamic size measurement of porcine pancreatic α-amylase in PBS buffer at pH 5.4, along with decaying autocorrelation function obtained from dynamic light scattering measurement with NanoZS (Malvern Instruments) (C) average pore size for triplicated measurements of each sample using gas sorption analysis against adsorption capacity of each sample (m_{tot}) at equilibrium, showing a sigmoidal transition from low uptake to high uptake. Markers with digits and letter are from individual measurements and analysis of MSP 1-Pristine (1P), MSP 1-M (1M), MSP 1-C (1C), MSP 2-Pristine (2P), MSP 2-C (2C), and MSP 2-H (2H).

References

[1] M. Thommes, K. Kaneko, A. V. Neimark, J. P. Olivier, F. Rodriguez-Reinoso, J. Rouquerol, K. S. W. Sing, *Pure Appl. Chem.* **2015**, *87*, 1051.

[2] J. Schindelin, I. Arganda-Carreras, E. Frise, V. Kaynig, M. Longair, T. Pietzsch,

S. Preibisch, C. Rueden, S. Saalfeld, B. Schmid, J. Y. Tinevez, D. J. White, V. Hartenstein, K. Eliceiri, P. Tomancak, A. Cardona, *Nat. Methods* **2012**, *9*, 676.
